# PREDICTING EXCESSIVE SPENDING IN OLDER ADULTS: THE IMPACT OF COGNITIVE STATUS AND MENTAL HEALTH

**DOI:** 10.1093/geroni/igae098.3690

**Published:** 2024-12-31

**Authors:** Emily Flores, Peter Lichtenberg

**Affiliations:** Wayne State University, Detroit, Michigan, United States; Wayne State University, Detroit, Michigan, United States

## Abstract

Excessive spending among older adults poses a significant risk to financial stability, particularly in the context of cognitive and psychological factors. This study utilized data from the WALLET study to examine predictors of excessive spending, focusing on sociodemographic variables, cognitive status, and mental health as measured by the Geriatric Depression Scale (GDS). The sample included 150 older adults, predominantly African American (65%) and female (80%), with an average age of 72.9 years (SD = 7.9). Correlation analyses found significant associations between excessive spending and cognitive status (r =.18, p <.05) and GDS scores (r =.21, p <.01). Regression analyses showed that in the first model, cognitive status (β =.18, p <.05) was a significant predictor of excessive spending when measured as a percentage, while education was also significant (β =.19, p <.05). However, the second model revealed that higher levels of depression (β =.21, p <.05) significantly predicted excessive spending, even after controlling for sociodemographic and cognitive status. These findings highlight the role of depression in financial risk behaviors, and it underscores the need for targeted interventions focusing on cognitive and psychological assessments to address excessive spending in older adults.

